# Reducing the effective dose of cisplatin using cobalt modified silver nano-hybrid as a carriers on MCF7 and HCT cell models

**DOI:** 10.1186/s13065-024-01173-8

**Published:** 2024-04-10

**Authors:** Amna H. Faid, Marwa A. Ramadan

**Affiliations:** 1 Department of Laser Science and Interaction, National Institute of Laser Enhanced Science (NILES) Cairo University, Giza, Egypt; 2 Department of Laser Application in Metrology, Photochemistry and Agriculture, National Institute of Laser Enhanced Science (NILES) Cairo University (CU), Giza, Egypt

**Keywords:** Core–shell, Cobalt–silver, Laser photostability, Drug delivery, Cisplatin, Colon carcinoma, Breast carcinoma

## Abstract

Cancer is a deadly illness with a convoluted pathogenesis. The most prevalent restrictions that frequently result in treatment failure for cancer chemotherapy include lack of selectivity, cytotoxicity, and multidrug resistance. Thus, considerable efforts have been focused in recent years on the establishment of a modernistic sector termed nano-oncology, which offers the option of employing nanoparticles (NPs) with the objective of detecting, targeting, and treating malignant disorders. NPs offer a focused approach compared to conventional anticancer methods, preventing negative side effects. In the present work, a successful synthetic process was used to create magnetic cobalt cores with an AgNPs shell to form bimetallic nanocomposites CoAg, then functionalized with Cis forming novel CoAg@Cis nanohybrid. The morphology and optical properties were determined by TEM, DLS, FTIRs and UV–vis spectroscopy, furthermore, anticancer effect of CoAg and CoAg@Cis nanohybrids were estimated using MTT assay on MCF7 and HCT cell lines. Our results showed that Co@Ag core shell is about 15 nm were formed with dark CoNPs core and AgNPs shell with less darkness than the core, moreover, CoAg@Cis has diameter about 25 nm which are bigger in size than Co@Ag core shell demonstrating the loading of Cis. It was observed that Cis, CoAg and CoAg@Cis induced a decline in cell survival and peaked at around 65%, 73%and 66% on MCF7 and 80%, 76%and 78% on HCT at 100 µg/ml respectively. Compared to Cis alone, CoAg and CoAg@Cis caused a significant decrease in cell viability. These findings suggest that the synthesized CoAg can be used as a powerful anticancer drug carrier.

**Graphical Abstract:**

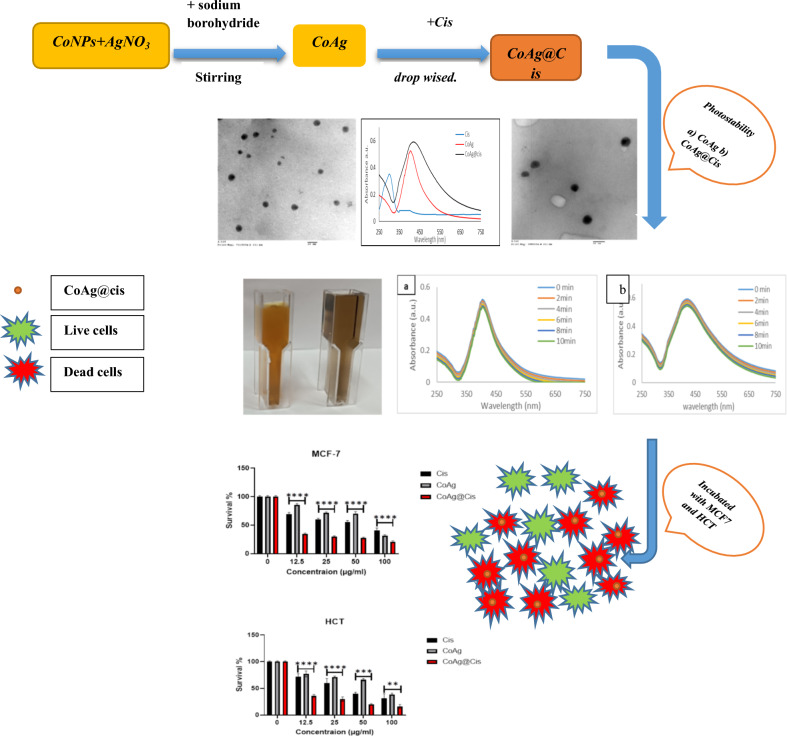

## Introduction

Cancer has an unfortunate prognosis due to its aggressiveness and lack of effective treatments, and its occurrence is rising at a startling rate. The optimal type of cancer therapy has long been sought after, but present therapies are inadequate because most lack sensitivity, specificity, and affordability. Due to the severe side effects of conventional anticancer therapy and the high concentration of therapeutic medications utilized, a treatment plan that maximizes the drug's effectiveness on cancer cells while minimizing its effectiveness on healthy, rapidly dividing cells is required. Recent research has focused on using nanoparticles to deliver anticancer drugs to cancer cells since angiogenesis makes tumors more permeable to these delivery systems. Cobalt (Co) transition metal compounds have strong antibacterial properties and anticancer activities [1–4]. Metallic nanoparticle-loaded magnetic cores could be effective nano-carriers for effective medicine delivery at infectious areas [5–8]. Silver nanoparticles (AgNPs), which are essentially one of the major qualities for good conductivity, chemical stability, relative decreased toxicity, and outstanding therapeutic potential are among the nanoparticles being explored [9–13]. It has also been investigated as a means of delivering therapeutic material to the nucleus, thereby focusing on sick cells [6, 14]. There are many factors that may have an influence on the biological activity of AgNPs, these factors include size distribution, morphology, surface charge, surface chemistry, capping agents [15–17].

Co has been used as the core material in a variety of nanocomposite materials, such as cobalt-gold (Co@Au), cobalt-copper (Co@Cu), cobalt–platinum (Co@Pt), and cobalt-silver (Co@Ag) nanoparticles. The key benefits of using Co as a core material Combined with other non-magnetic materials like Ag, Au, and Cu, were the magnetoresistance, excellent stability even at greater temperatures, both of which are desired qualities in multipurpose applications. Numerous Bimetallic hetero-nanostructures are created through chemical, physical, and biological methods; However, because it is easy to handle, chemical reduction technique might be beneficial cost efficiency and gives excellent quality, purity, thermal stability and particle size adjustment [18–21]. Clinical studies have demonstrated the effectiveness of conventional chemotherapeutic medications like Cisplatin (Cis) in treating a variety of cancers, including sarcomas and tumors of the soft tissues, bones, muscles, and blood vessels [22–24].

As the first platinum-based medication to receive US-FDA approval, cisplatin [cis-diamine platinum (II) dichloride] is frequently used as the drug of choice for treating a variety of cancers [5, 6, 25]. Through a variety of biochemical pathways, cisplatin interacts with cellular macromolecules and causes cytotoxicity by binding to DNA and creating intra-strand DNA adducts that prevent DNA synthesis and cell development [26]. Due to their potential use in numerous biomedical applications, metallic nanoparticles' distinctive optical, electrical, and biological characteristics have drawn substantial attention [27–29]. controlled release, drug targeting, and much higher bioavailability of medications are all features of nanodrug delivery systems which greatly overcome the weaknesses of traditional drug delivery [30–34]. According to earlier research, poly-nano-complexes and anticancer medications can boost the anticancer drug accumulation in tumor cells for a more effective treatment outcome [35–37]. AgNPs exhibit a synergistic effect and a cytotoxic effect on cell viability [13], which play a key part in the antitumor action. AgNPs help collect and deliver medications into cancer cells, and they also prevent cancer metabolism and tumor growth. According to earlier research, AgNPs can cause cell death both in vivo and in vitro by an apoptotic mechanism that is driven by reactive oxygen species (ROS) [35, 38–40]. Here, we investigate the formation of a novel biocompatible nanodrug by combining CoAg and Cis in order to increase the therapeutic index on MCF7 and HCT Cell Lines.

## Material and methods

### Preparation of CoAg nanohybrid

In the First step cobalt nanoparticles (CoNPs) prepared by metal salt reduction method in which 1 gm poly vinyl alcohol (PVA) dissolved in 20 ml warm water after complete dissolve 3 ml (0.05 M) Co were added with continuous stirring for 15 min. Then 5 ml (0.05) sodium borohydride drop wised with stirring until become completely dark. The second step is coating CoNPs with AgNPs as a way to stabilize the CoNPs because of their relative low stability in air due to the smaller size [41]. The CoAg were prepared by reducing silver nitrate in the presence of pre-synthesized CoNPs which act like ‘‘seeds’’ or nucleation sites for the resultant CoAg [1, 2]. In this step 7 ml of the prepared CoNPs were stirred with 7 ml (0.05M) AgNO_3_ for 15 min in dark conditions, then 5 ml (0.05 M) sodium borohydride was added into the flask drop wise under the stirring this slowly injection to avoid mass production of pure AgNPs. A dark yellowish color results from the reduction process and denotes the creation of CoAg [20].

### Preparation of CoAg@Cis

In this experiment 5 ml of 0.1 mg/ml Cisplatin was added drop wised to 5 ml of the prepared CoAg sample with continuous stirring for 30 min in dark conditions.

### Photostability of CoAg and CoAg@Cis

The photostability of CoAg and CoAg@Cis have been studied by irradiation with light emitting diode (LED), (490 nm and 250 mW). Before exposure, the solution's absorption spectra were measured, and then it is irradiated with the light source. To track any spectrum changes following irradiation, the absorption spectra have been monitored at various time intervals.

### Characterization of CoAg and CoAg@Cis

UV–visible absorbance spectra were measured using a double beam spectrophotometer (PG instrument, T80 + , UK). 300 µl from (CoAg, Cis and CoAg@Cis) were diluted to 3 ml with distilled water and absorption has been recorded. For each sample the spectra have been compared with distilled water as a reference. Transmission electron microscope JEOL (JEM-1400 TEM) and TEM lab FA-CURP, Faculty of Agriculture, Cairo University Research Park, were used to examine the morphology of the produced solutions. Shimadzu FT-IR 8400 FT-IR spectrometer was used to do IR measurements in the 500–4500 cm^–1^ range. CoAg, Cis, and CoAg@Cis prepared samples were dried with a lyophilizer. A potassium bromide (KBr) pellet was used to dilute the IR spectra of the powdered materials. The XRD analysis was done using a Bruker AXS D8 Advance x-ray diffractometer with a CuKα source at rate 5°. Using a DLS system and a Zeta sizer 300 HAS (Malvern Instruments, Malvern, UK), the particle size and surface charges of CoAg and CoAg@Cis were examined. The average zeta potential was calculated following a 60-s analysis. Without using any dilution, the zeta potential of the nanoparticulate dispersion was established.

### Cytotoxicity assay

Cell line purchased from National Cancer Institute (NCI), Cairo, Egypt and was preserved as “monolayer culture” using RPMI medium supplemented with 10% FBS and 2% Pen/Strep. Cells were incubated at 37 °C in 5% CO_2_ in a high humidity atmosphere in a water jacketed incubator (Thermo Fisher Scientific USA). The lines were repetitively sub-cultured to be kept in the exponential growth phase. Sterile conditions were achieved by working under an equipped laminar flow (Microflow Laminar flow cabinet, MDH limited, Hamsphire SP105AA, U.K.). Cells were grouped into control group and treated groups with different concentration (12.5, 25, 50, and 100 µg/ml) of Cis, CoAg, CoAg@Cis. After 48 h, add 10 μl of the MTT reagent (concentration 0.5 mg/ml) to each well. Incubate the microplate for 4 h. Add 100 μl of the Solubilization solution into each well. After complete solubilization of the purple formazan crystals, measure the absorbance of the samples using a microplate (ELISA) reader. The wavelength to measure absorbance of the formazan product is570 nm. The cell viability percentage was calculated using the following equation:

$${\text{The cell viability }}\left( \% \right) \, = \, \left[ {{\text{ODS}}/{\text{ ODC}}} \right] \, \times { 1}00.$$

where, ODS stands for the sample’s mean optical density, while ODC is control's mean optical density.

### Statistical analysis

The results were displayed by a graph of percentage of cell viability versus the concentrations of the tested materials using. Graphpad Prism 8.4.3 statistical analysis program was used to compile the data. Tukey's multiple comparison test was used for statistical analysis of transfection assay results and the data obtained as a mean ± standard deviation. Significant differences were defined as those with a probability p ≤ 0.05.

## Results and discussion

Synthesize of CoNPs, with narrow size distributions and controlled properties, have strong impact on the development of magnetic sensors and other biomedical applications. CoNPs are usually synthesized by the reduction of Cobalt salts [42, 43].

In this work we have thought about the synthesis of CoNPs in water-based solution and the method was achieved by the reduction of the metal salt with strong reducing agent in the presence of a long chain surfactant PVA (poly vinyl alcohol) polymer of predetermined concentration to ensure the high colloidal stability, applicable in biological applications and also the method achieves the advantage of the simple and cheap chemistry [44, 45]. The polar functional groups of the polymer are deducted to introduce a considerable hydrogen bonding between the surface of the CoNPs and the polymer chains and also may be responsible for the cross linking between the polymer chains as well. The synthesis procedure for CoAg consists of two stages: the first is the reduction of cobalt ion, and the second is the reduction of silver ions in the presence of prepared CoNPs as seeds, this process can be outlined by the following Equations.

$${\bf{4Co}}^{{\bf{ + 2}}} {\bf{ + NaBH}}_{\bf{4}} {\bf{ + 3H}}_{\bf{2}} {\bf{O \to 4Co}}^{\bf{0}} \left( {{\bf{metal}}} \right){\bf{ + Na}}^{\bf{ + }} {\bf{ + B}}\left( {{\bf{OH}}} \right)_{\bf{3}} {\bf{ + 7H}}^{\bf{ + }} {\bf{.}}$$

$${\bf{NaBH}}_{\bf{4}} {\bf{ + 4 H}}_{\bf{2}} {\bf{O \to NaOH + B}}\left( {{\bf{OH}}} \right)_{\bf{3}} {\bf{ + 4H}}_{\bf{2}} \left( {{\bf{gas}}} \right){\bf{.}}$$

Then, silver ions are reduced by hydrogen gas which is produced by the hydrolysis of sodium borohydride. This led to formations of Ag atoms which diffuse to Co metals and form CoAg as shown of our previous work [20].

**Fig. 1:**
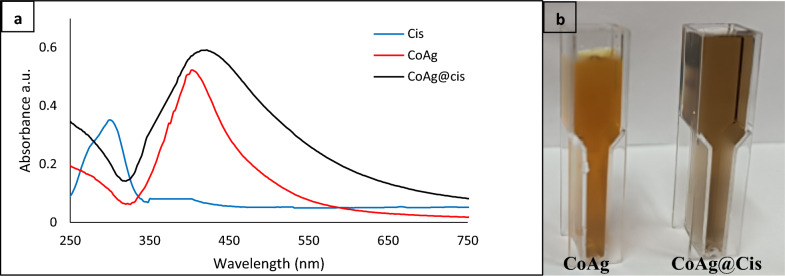
**a** Absorption spectra of Cis, CoAg, CoAg@Cis (**b**) digital photograph of CoAg, CoAg@Cis

These CoAg in aqueous solution are stable up to 10 months without the observation of cobalt oxide. Another evidence for the presence of bimetallic nanocomposites comes from the optical absorption study of each case. The appearance of the plasmon band for the AgNPs implies that silver metal coexists with the CoNPs. In Fig. 1a, the absorption peak of Cis is at about 302 nm, hence Ag NPs, in a previous researches has a sharp absorption peak around 400 nm [35–37] the absorption curve of Co@Ag, shows a sharp peak at 410 nm which indicates the formation of Ag shell on the surface of Co nanoparticle core, upon Cis addition on CoAg core shell, broadening and slight red shift from 410 to 425 nm for the CoAg@Cis nanohybrid which indicate successful conjugation of Cis to CoAg. In (Fig. 1b), CoAg, CoAg@Cis solutions appear to be uniform and CoAg@Cis much darker in color compared with the CoAg which strongly suggested the incorporation of Cis with CoAg and forming CoAg@Cis.

**Fig. 2:**
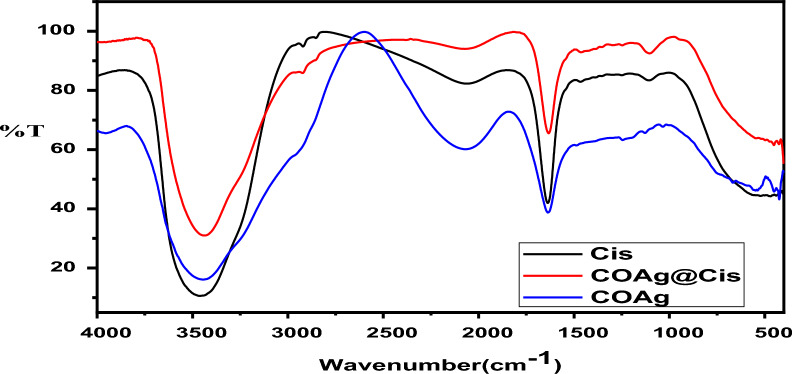
FTIR spectra of Cis, CoAg, CoAg@Cis

CoAg@Cis nanohybrid was examined using FT-IR studies in Fig. 2, the IR spectra of free Cis, CoAg and CoAg@Cis are shown in Fig. 2, Cis solution show bands at 3.460 cm^−1^, 2.922 cm^−1^, 1.638 cm^−1^ and 1.105 cm^−1^ corresponding to O–H stretching, aldehydic C–H stretching, C–N stretch vibration, and O–H stretch respectively. [46] And FTIRs for CoAg nanohybrid show bands in the region between 3953 cm^−1^ to 3448 cm^−1^ were assigned to O–H stretching and–C–H– stretching of alkanes. The peaks 1637 cm^−1^ correspond to C–N stretch vibration, bands from 1244 cm^−1^ to 1034 cm^−1^ correspond to primary and secondary amides of N–H (bond) of and –C–N– stretching vibration of amines [47]. In CoAg@Cis, a decrease in intensity of all bands were observed and O–H stretching and–C–H– stretching of alkanes become sharper with blue shift from 3448 cm^−1^ to 3439 cm^−1^. In addition, there were slightly blue shifts for C–N stretching to1632 cm^−1^. The free –NH group may be involved in the binding of Cis on the CoAg confirming the successful conjugation of Cisplatin on CoAg nanohybrid [30].

**Fig. 3:**
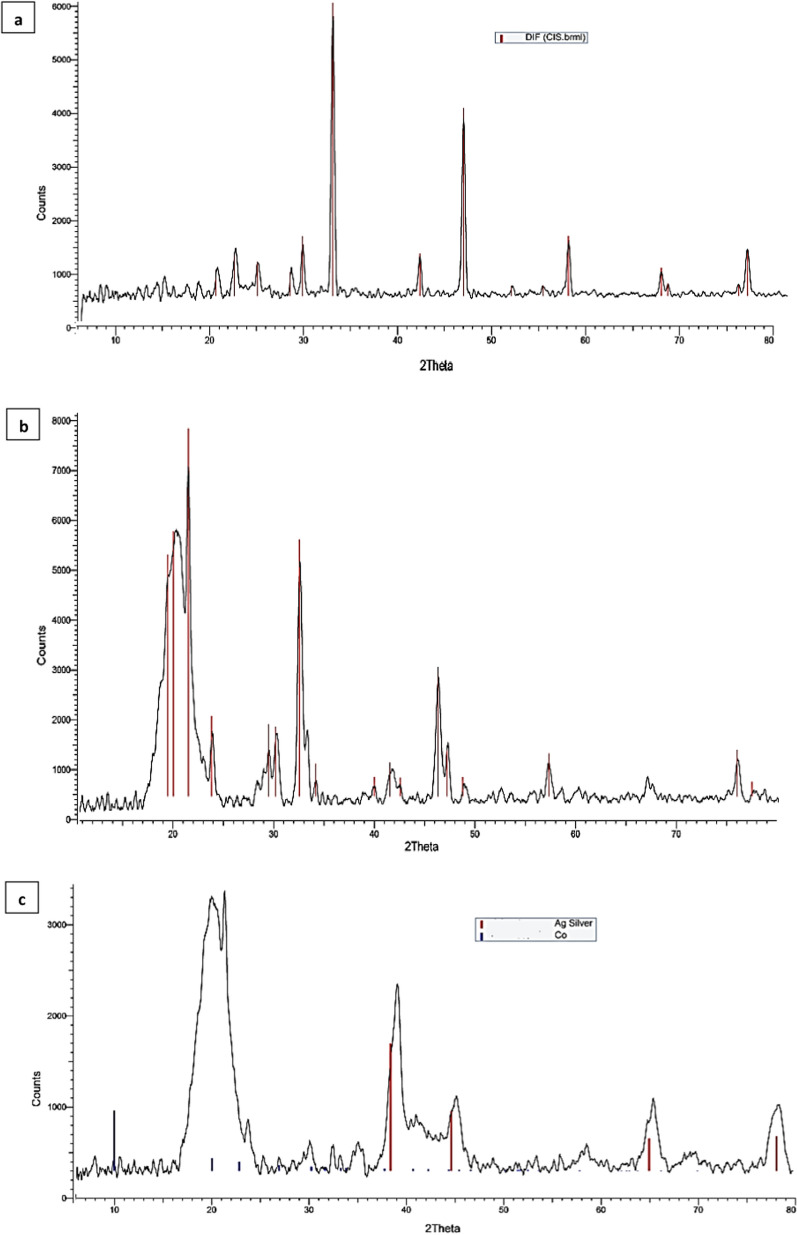
X-ray diffraction (XRD) patterns of (**a**) Cis, (**b**) CoAg, (**c**) CoAg@Cis

Crystallinity is one of the major factors that affect the mechanical properties of nanoparticles and nanocomposites. As shown in Fig. 3 The structures of the synthesized products were characterized using powder X-ray diffraction (XRD) patterns diffractometer (Bruker; model D8 Advance) with monochromatic Cu-K radiation (1.54178 Å). The sharp peaks appeared in the Fig. 3a at 2θ = 27, 30.1, 36.7, 44.1, 48.5 and 57 degrees due to the platinum in the Cis. [48] The characteristic peaks for the Ag was observed in Fig. 3b at 2θ = 30°, 35.5°, 43°, 54°, 57° and 63° of planes (111), (200), (220) and (311) of FCC with an average crystallite size of 8 nm.^[49]^Also The characteristic peaks of Co was observed at 2θ = 44° of plane (111) with low intensity due to it is a core and covered with a shell of Ag. [50] This overlapping between the two peaks indicate the bimetallic crystalline nature of the CoAg. Also, The XRD pattern of the PVA revealed strong crystalline reflections at around 2θ = 19.92° and a shoulder at 22.74°. The two peaks are characteristic of PVA, representing reflections from (101) and (200). [51] The CoAg@cis sample in (Fig. 3c) shows a broad PVA peak at 2θ = 19.92° and a shoulder at 22.74° and other peaks at 27, 30.1,36.7, 44.1, 48.5 and 57 degrees corresponding to the Cis. As appeared in Fig. 3c the Cis peaks appear with the interference with CoAg at 2θ = 10–25 degrees, this is because Cis to CoAg ratio in the composite is very small, so the XRD confirmed that these results were strong evidence for successful cisplatin loaded into CoAg nanohybrid.

**Fig. 4:**
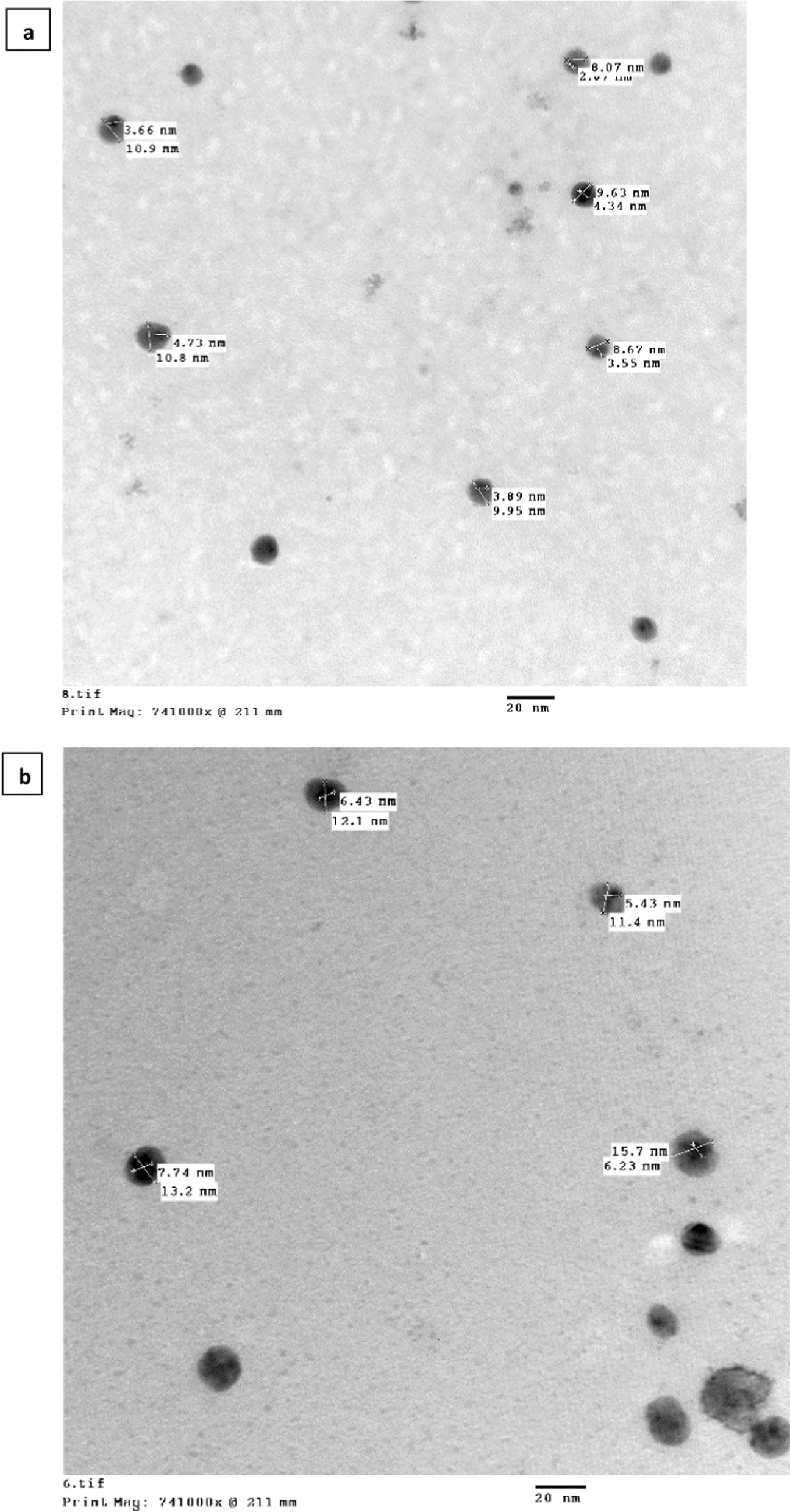
TEM Images of (**a**) CoAg (**b**) CoAg@Cis (magnification 20 nm)

In Fig. 4a, the TEM image exposes that Co@Ag core shell with diameters at about 10 nm were formed with dark CoNPs core diameters at about 4 nm and AgNPs shell with less darkness than the core. Figure 4b, shows TEM image of CoAg@Cis with diameters at about 15 nm which are bigger in size than Co@Ag core shell demonstrating the loading of Cis.

**Fig. 5:**
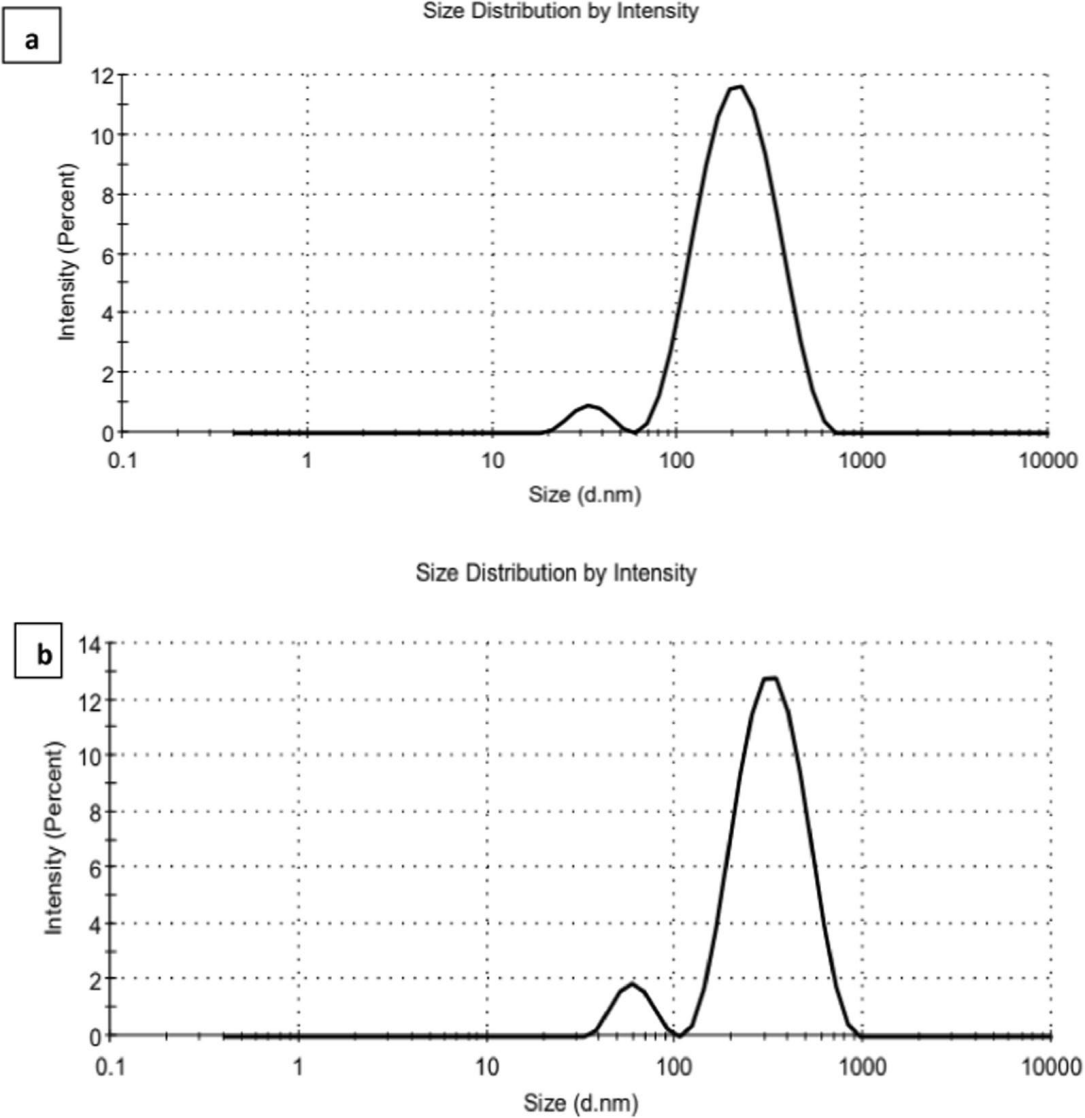
Particle size of (**a**) CoAg (**b**) CoAg@Cis

**Fig. 6:**
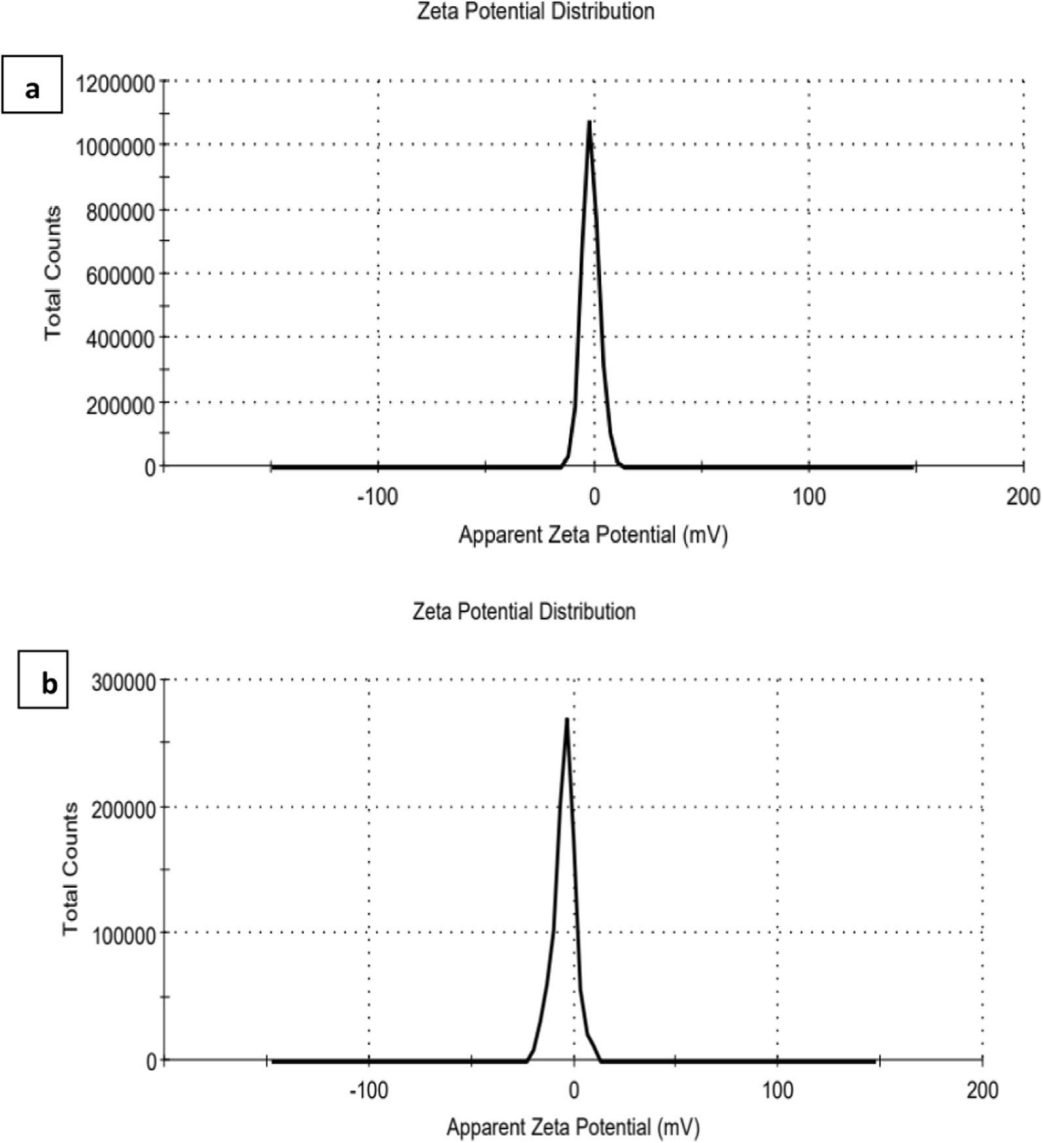
zeta potential of (**a**) CoAg (**b**) CoAg@Cis

DLS results revealed that CoAg before and after conjugation process had different sizes and disparities which confirmed the changes in the surface of the CoAg due to the CoAg@Cis formation. As it can be seen in Fig. 5a, b the average size of the CoAg after conjugation with Cis drug increased from172.5 nm to be 242 nm. Also, zeta potential of the CoAg was − 1.7 mV and it becomes − 4.6 mV after the conjugation of Cis and forming CoAg@Cis which is more stable for about 10 months as it can be seen in Fig. 6a, b and Table 1.

**Table 1:** The parameters of CoAg and CoAg@Cis

	CoAg	CoAg@Cis
Average zeta potential	− 1.7 mv	− 4.6 mv
Average hydrodynamic diameter (DLS)	172.5 nm	242 nm
Average Size (TEM)	10 nm	15 nm

**Fig. 7:**
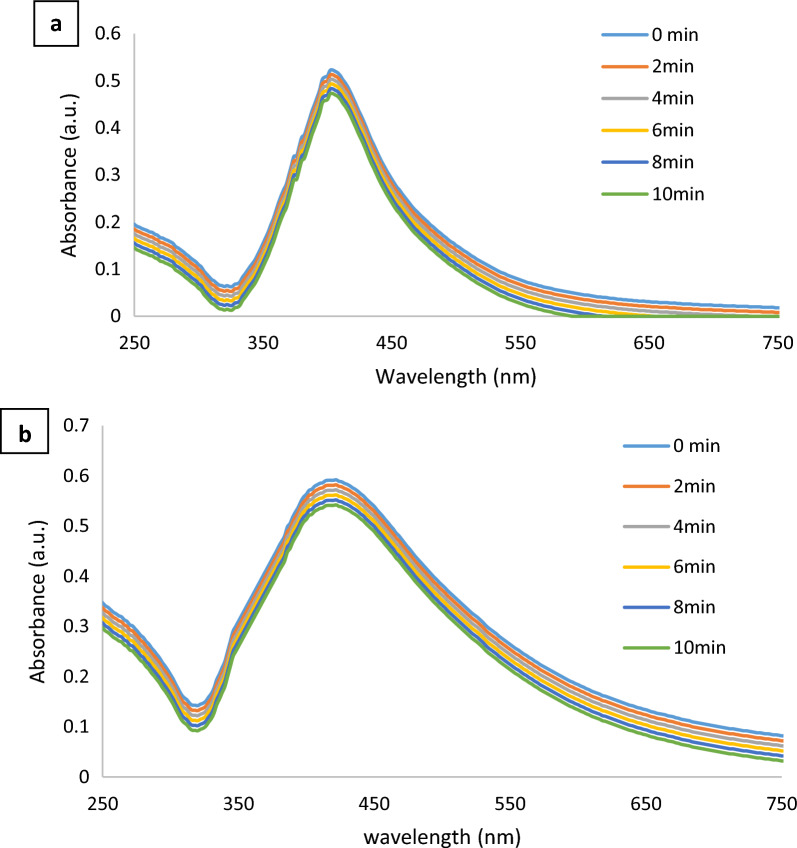
Effect of irradiation by LED on (**a**) CoAg (**b**) CoAg@Cis at different exposure time

CoAg and CoAg@Cis are promising materials which can be used in photothermal therapy (PTT) for cancer, which highlights the importance of the requirement for photostability. When it is decided to use these particles as PTT hyperthermic agents, the target tissue or cells will be subjected to laser light. By exposing CoAg and CoAg@Cis to the same LED light source for the same exposure period, we must evaluate the stability of these two materials. Surface Plasmon Resonance (SPR) of the exposed CoAg and CoAg@Cis is unaffected by the LED light source, as shown in Fig. 7a, b. This indicates that the produced nanomaterials are photothermally stable, and they will be effective in PTT [12].

### In vitro cytotoxicity of Cis, CoAg and CoAg@Cis.

**Fig. 8:**
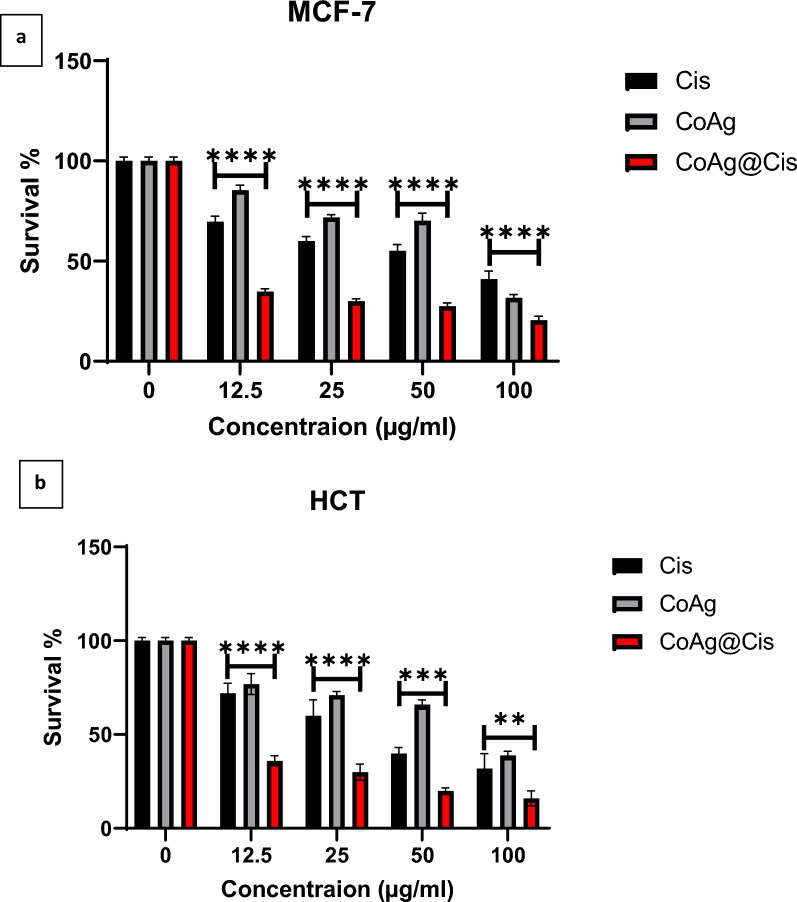
toxicity test at different concentrations (µg/ml) of Cis, CoAg, and CoAg@Cis on (**a**) MCF-7 (**b**) HCT cell lines

MTT assay was used to determine if the nanomaterials were biocompatible in vitro at various concentrations. Exponentially dividing cells were treated with increasing concentrations (12.5, 25, 50, and 100 µg/ml) of Cis, CoAg, and CoAg@Cis on MCF7 and HCT cell lines. Our results were in accordance with previously reported work, as shown in Fig. 8a, in comparison to the corresponding control, there was a concentration-dependent decline in cellular proliferation. Cis, CoAg, and CoAg@Cis revealed a significant decrease in cell viability. It is shown with IC50 value of about 71.2%, 76% and 6.5% on MCF7 and 38.2%, 78%and 8.7% on HCT respectively. Comparing CoAg@Cis to native Cis we can observe that CoAg@Cis nanohybrid gives very high significant inhibition on both MCF7 and HCT cell lines as in Table 2 [1, 52].

**Table 2:** IC50 values for Cis, CoAg and CoAg@Cis on MCF-7 and HCT cell lines

Cell lines	Cis IC50 (µg/ml)	CoAg IC50 (µg/ml)	CoAg@Cis IC50 (µg/ml)
MCF-7	71.2	76	6.5
HCT	38.2	78.5	8.7

The ability of cisplatin to crosslink with DNA's purine bases has been credited as the drug's mechanism of action. This crosslinking prevents DNA from being repaired, damages DNA, and kills cancer cells. Cisplatin interacts with cellular macromolecules through several pharmacological mechanisms. It then causes death by attaching to DNA and creating intra-strand DNA adducts that prevent DNA synthesis and cell growth. Its primary molecular mechanism of action has been linked to the activation of various signal transduction pathways, induction of p53 signaling and cell cycle arrest, upregulation of pro-apoptotic genes/proteins, and downregulation of proto-oncogenes and other tumor-promoting genes as a result of the production of reactive oxygen species through lipid peroxidation [26].

The surface of cancer cells is negatively charged because of the release of lactic acid, according to extensive research. As a result, the concentration, hydrophilicity, surface charge, and size of the nanomaterial may affect the endocytosis of the nanoparticles. Additionally, the different cell types consume nanomaterials in different ways [53]. The improvement brought about by CoAg@Cis nanohybrid's internalization by an endocytosis mechanism is one explanation for the elevation of its activity. Comparing the passive diffusion method of free Cis into cells to that of nanoparticles, endocytosis or phagocytosis typically results in the nonspecific internalization of nanoparticles into cells [54].

## Conclusion

In conclusion, we have reported the synthesis of novel theragnostic nanohybrid agent for cisplatin delivery systems. The results of this study dispense basic information for synthesizing CoAg and CoAg@Cis nanohybrid and their anticancer effect on MCF7 and HCT cell lines. Our results revealed that CoAg@Cis nanohybrid were successfully formed as indicated from broadening and red shift in absorption band of CoAg after Cis addition to increase in particle size, moreover the enhanced cytotoxic effect of CoAg@Cis nanohybrid than free Cis is an indication that CoAg can be used as drug carrier. CoAg@Cis nanohybrid has more inhibition with IC50 value decreased to 8.7 µg/ml on HCT and 6.5 µg/ml on MCF7. Future work is to use the prepared nanohybrids for photothermal chemotherapy combine treatment for in vitro and in vivo treatment.

## Data Availability

The datasets used and/or analyzed during the current study are available from the corresponding author on reasonable request.
